# Neuro-Arthropathy in a Schizophrenic Patient

**DOI:** 10.5334/jbsr.2124

**Published:** 2020-06-22

**Authors:** Sylvain Guillaume, Frederic Lecouvet

**Affiliations:** 1 Cliniques universitaires Saint-Luc, BE

**Keywords:** Neuro-arthropathy, pain sensation, schizophrenia, knee, diabetes

## Abstract

Neuro-arthropathy is a destructive osteo-articular condition with typical radiological features that can be the result of every disease affecting the sensitive nervous pathways.

## Case

**Figure 1:**
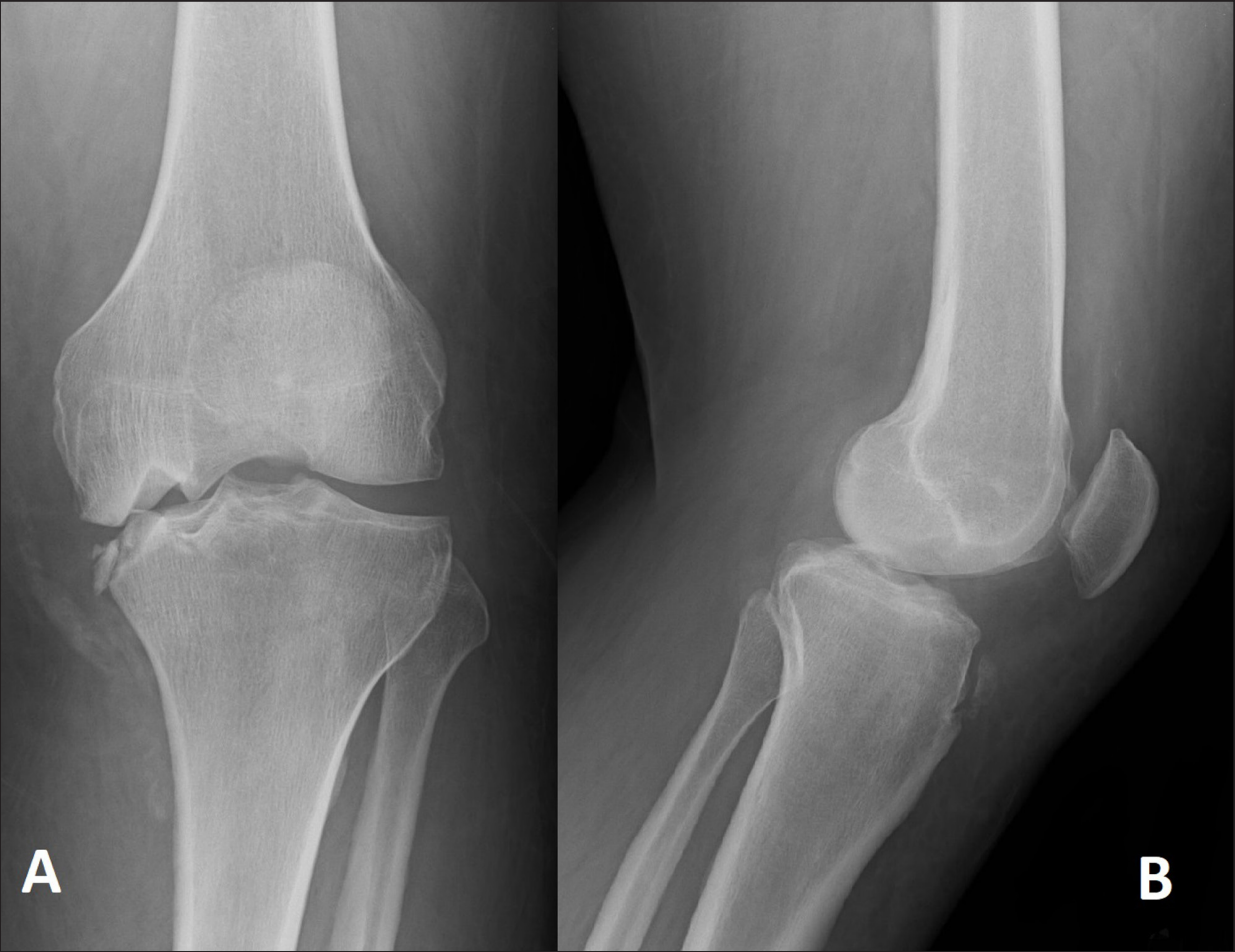


**Figure 2:**
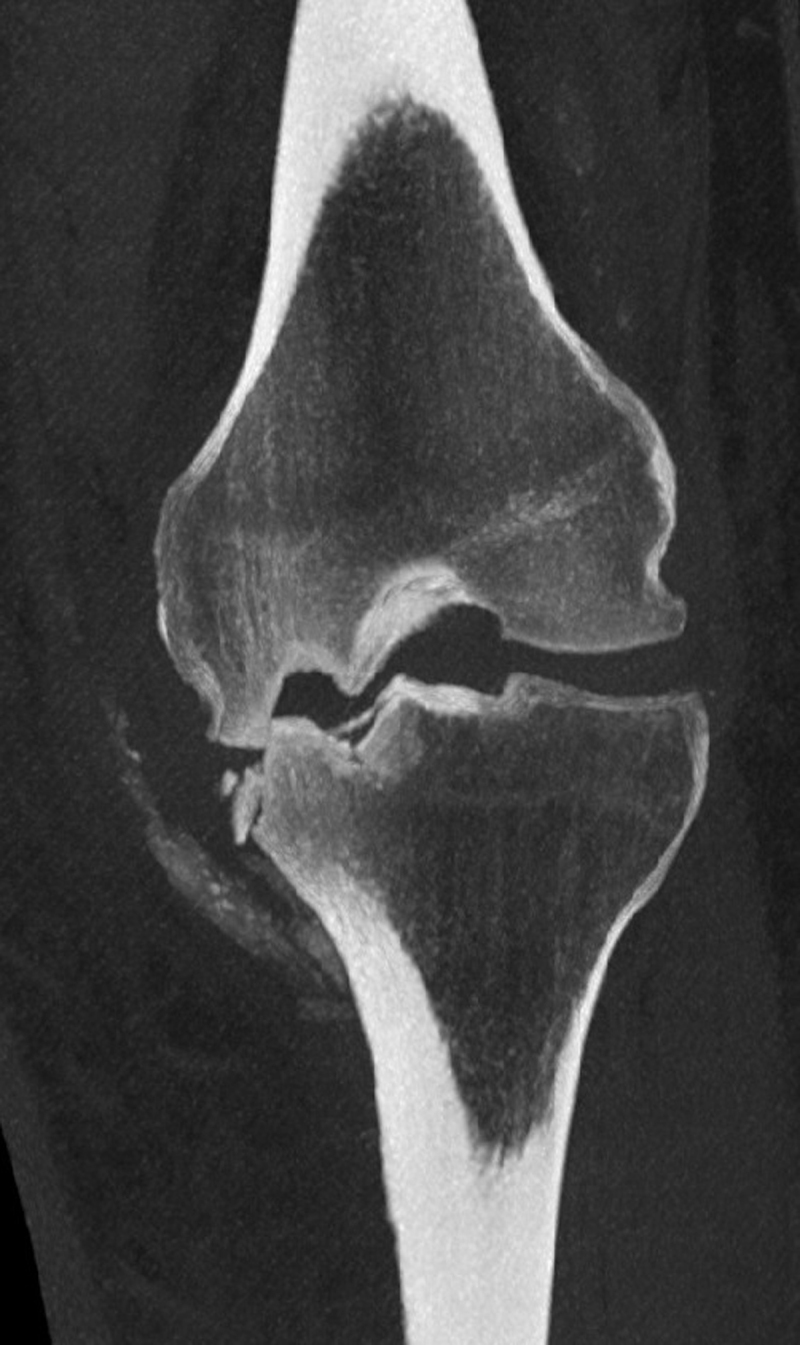


**Figure 3:**
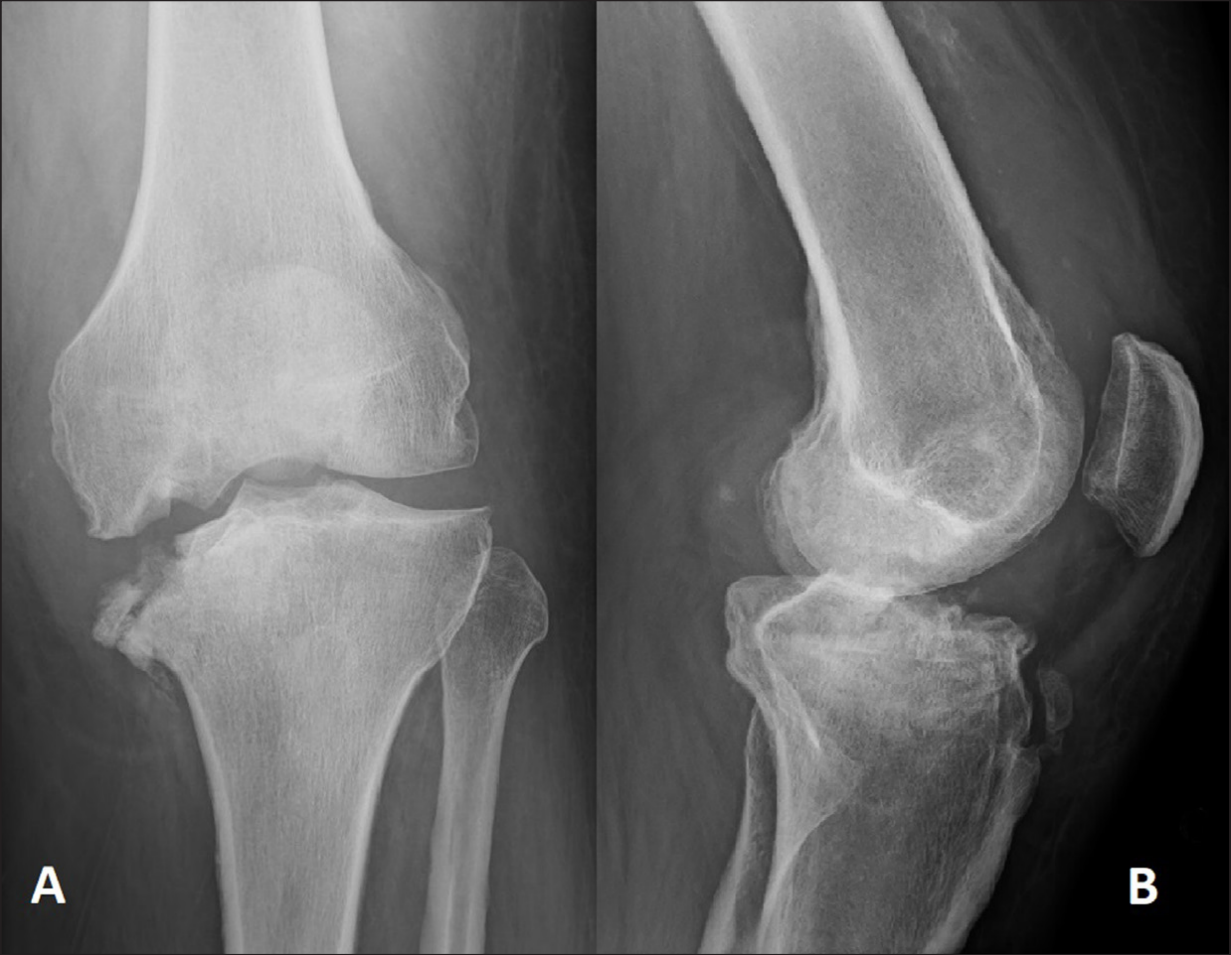


A 45-year-old man came to the emergency service exhibiting non-traumatic swelling and moderate pain in the left knee. The joint was red and hot at physical examination, and the neurologic tests were normal. He had a medical history of schizophrenia (treated with Olanzapine and Quietapine), diabetes (neuroleptic induced, well balanced with Metformine), alcoholism (no B12 deficiency), and obesity (Body Mass Index = 47). Biology was normal except for moderate systemic inflammation (C-Reactive protein 68.1 mg/L [<5 mg/L]). Left knee radiographs showed impacted medial femoro-tibial articular surfaces with “mirror deformity”, sub-chondral sclerosis, bone fragmentation with intra-/peri-articular calcified debris (Figure 1A) and joint effusion (Figure 1B). There were no erosions nor osteophytes. Computed tomography (CT) (Figure 2) confirmed these observations. At this stage, a septic/inflammatory osteoarthritis could reasonably be excluded. A classic fracture was also unlikely. Thus, the final retained diagnosis was neuro-arthropathy. Follow-up radiographs at four months showed a progression of the articular space collapse, deformity, and fragmentation (Figure 3A), a persistent joint effusion with (peri-)articular calcified debris (Figure 3B). More important, there was no sign of consolidation.

## Comment

Neuro-arthropathy is an osteo-articular disorder of uncertain pathogenesis. It is believed that a neuropathic background can result in a major joint destruction due to the loss of pain sensation. The initial injury (micro/macro-trauma), going unnoticed, leads to inflammatory response, unnoticed too, which results in bone surface alteration that favors new injuries. This vicious circle ends in severe joint destruction, with or without bone reformation (hypertrophic or atrophic forms). At the early stage of the disease, the joint presents swelling, redness, and heat, but the radiographic abnormalities are very subtle (sub-chondral bone rarefaction, joint effusion, minor sub-luxation, etc.) and the diagnosis is often missed. Magnetic resonance imaging (MRI) can show medullar edema and is useful to rule out septic complication, especially in the foot (Charcot). A period of rest and avoiding weight bearing can stop the progression to the chronic stage. At this point, the radiographic abnormalities are evident, and can be summarized by the 6 Ds mnemonic: density change (osteopenia/sclerosis), destruction (fragmentation), debris (loose bodies), distension (effusion), disorganization, and dislocation. The treatment can be conservative or surgical. The far most common cause in our modern society is long-standing and uncontrolled diabetes, especially in patients with peripheral neuropathy. By extension, every disease that affects the peripheral or central nervous sensitive pathways can lead to neuro-arthropathy; that includes alcoholism (B12 deficiency), syrinx medullary trauma, and the like.

In the absence of any of these more typical causes, the hypothesis of schizophrenia was retained as the cause of this knee neuro-arthropathy. Indeed, this condition is known to affect the pain sensation, with a higher pain threshold. Some schizophrenic patients may suffer from congenital insensitivity to pain [1].
